# The influence of core-build up materials on biaxial flexural strength of monolithic strength-gradient zirconia; an in-vitro study

**DOI:** 10.1186/s12903-023-03635-2

**Published:** 2023-11-17

**Authors:** Dina B. Musa, Nadia S. Ereifej

**Affiliations:** 1 Amman, Jordan; 2 Department of Prosthetic Dentistry, Faculty of Dentistry, The University of Jordan, Queen Rania Street, Amman, 11942 Jordan

**Keywords:** Biaxial flexural strength, Bulk-Fill composites, Monolithic zirconia, Multilayered zirconia, All-ceramic restorations

## Abstract

**Background:**

Since their introduction, there has been limited research regarding the mechanical properties of novel strength-gradient monolithic zirconia. In addition to that, studies evaluating the effect of different core-build materials on the strength of indirect restorations are scarce. Therefore, the aim of this study was to investigate the effect of using different core build-up materials on biaxial flexural strength of a new monolithic multilayered zirconia material.

**Methods:**

Forty zirconia discs were fabricated from IPS e.max ZirCAD Prime (Ivoclar Vivadent AG, Schaan, Liechtenstein) and divided into 2 groups (*n* = 20). Forty composite discs were prepared from Tetric N-Ceram (Ivoclar Vivadent AG, Schaan, Liechtenstein) and MultiCore Flow (Ivoclar Vivadent AG, Schaan, Liechtenstein). The zirconia discs were adhesively cemented to the 2 types of composite forming 2 groups (Zirconia-Tetric N-Ceram and Zirconia-MultiCore Flow). Biaxial flexural strength was determined using a piston-on-3-ball test. The data were statistically analyzed with an independent t-test for significant differences (*p* = 0.05).

**Results:**

Tetric N-Ceram had significantly higher strength than MultiCore Flow (*p* < 0.001) but no statistically significant differences were found in strength values between Zirconia-Tetric N-Ceram and Zirconia-MultiCore Flow bilayered samples (*p* = 0.27).

**Conclusions:**

It was concluded that although the tested composite core materials significantly differ in their biaxial flexural strength values, they had no influence on the biaxial flexural strength of the overlying zirconia.

## Background

Despite the increased awareness of the importance of dental and oral health, the need for endodontic treatment is still high [1]. Several factors can affect the long-term survival of endodontically treated teeth such as coronal seal, the number of remaining cavity walls, the presence of ferrule, the placement of post and the use of indirect restorations [2, 3].

Core restorations used for endodontically treated teeth should restore the normal anatomical form of teeth in addition to improving the compressive and tensile strengths to resist functional and para-functional stresses [4]. Different direct core build-up materials are available among which composite resins are the most popular materials as some demonstrated outstanding mechanical properties for core build-ups [4–6]. Because of the problems faced with conventional composite resins when applied in increments such as voids and gaps, bulk-fill composite resins were introduced [7–9]. These composites have lower post-gel shrinkage, higher reactivity to light polymerization and penetration, in addition to their superior physical and mechanical properties [7, 10, 11]. Bulk-fill resin composites can be placed in increments of 4–5 mm thickness and photopolymerized in one step which saves the restorative procedure time and facilitates handling [12]. Compared to the conventional flowable and paste composite materials, bulk-fill composites were found to have comparable mechanical properties and survival rates [13–15].

Following the direct restorations, full coverage indirect restorations are recommended for severely damaged teeth with large structural loss to provide cuspal protection for endodontically treated teeth [16]. Different restorative materials such as gold, metal-ceramic, and all-ceramic materials have been successfully used, among which all-ceramic restorations offer superior aesthetics, resistance to corrosion, the ability for etching and bonding, and biocompatibility [17, 18]. Different all-ceramic materials are available nowadays including glass ceramics such as leucite and lithium disilicate ceramics and polycrystalline ceramics like zirconia [19].

The first generation of dental zirconia (3 Y-TZP) was the high-strength tetragonal crystalline phase, stabilized with 3 mol% yttria and enhanced with 0.25% alumina and used as core material [19]. Due to its poor translucency, this type of zirconia needs to be veneered by a thick layer of felspathic porcelain using the traditional layering technique, press-on technique or CAD/CAM technology [20–22]. Later on, monolithic zirconia was introduced in an attempt to overcome chipping and delamination problems associated with bilayered zirconia offering the advantages of having high flexural strength and satisfactory aesthetics, in addition to the reduction in production time needed in the laboratory and clinic [23, 24]. These are produced by increasing the percentage of yttria to 5 mol% which results in improved translucency but with inferior flexural strength values than 3 Y-TZP [25]. More recently, the yttria content was reduced to 4 mol% providing an intermediate composition between high strength 3Y-TZP and high translucency 5Y-TZP zirconia, and another newer generation was produced (4Y-TZP) [26]. Later on, pre-shaded polychromatic multi-layered zirconia systems have been developed which have the same generation of zirconia within each blank with no difference in the flexural strength values between the incisal and cervical layers [27–29]. These allow obtaining the shade-gradient of natural teeth where the highest translucency is in the incisal or occlusal region while the cervical area has increased chroma and opacity [28]. More recently, another multilayered technology has been introduced with the development of new strength-gradient zirconia materials combining different generations of zirconia together in one blank [27]. IPS e.max ZirCAD Prime (Ivoclar Vivadent, Schaan, Liechtenstein) blanks consist of layers of different zirconia materials, with smooth transition between the different layers, with 9-mm-thick high strength (> 1200 MPa) 3Y-TZP dentin layer at the bottom side of the blank forming the cervical part of the restoration, a 4-mm-thick 4Y-TZP transition layer, and a 3-mm-thick reduced strength (< 650 MPa) 5Y-TZP incisal layer at the top of the blank for improved translucency where the incisal and occlusal part of the restoration is formed [30, 31].

Flexural strength is a significant characteristic of brittle materials like ceramics due to its ability to determine their durability and longevity [32]. Biaxial flexural strength tests were developed and are used frequently to determine the fracture characteristics of ceramics [33]. Compared to uniaxial flexural tests, biaxial flexural strength tests are more reliable since the maximum tensile stresses occur within the central loading area making them less sensitive to the edge effects and surface imperfections [34–36]. Since biaxial flexural stress reproduces multiaxial stress conditions in real applications without favoring crack in a particular direction, the biaxial flexural strength test was recommended by the American Society for Testing and Materials as an international standard for evaluating dental ceramics [33]. Several biaxial flexural strength tests have been developed including piston-on-ring, piston-on-three-ball, ball-on-ring, and ring-on-ring tests [34]. According to The International Organization for Standardization (ISO), the piston-on three-ball test has been adopted to establish ISO 6872:2019 for dental ceramics as this test is less sensitive to undetectable surface flaws at the loaded position which facilitates the accommodation of slightly warped specimens and it is not affected by the presence or absence of frictional contact between the three supporting balls and the disc-shaped specimen [33, 37].

Limited number of studies have been undertaken to evaluate the clinical performance of novel strength-gradient monolithic zirconia and studies evaluating the effect of different core-build materials on the strength of indirect restorations are scarce. Therefore, the aim of this study was to investigate the effect of using different composite core build-up materials on the flexural strength values of new highly translucent zirconia materials. The null hypotheses tested were that there is no statistically significant difference in biaxial flexural strength values between monolithic strength-gradient zirconia restorations cemented to 2 different composite core materials and that there is no statistically significant difference in values of biaxial flexural strength of the 2 composite core build-up materials tested.

## Methods

Forty bilayered disc-shaped specimens fabricated from monolithic strength-gradient zirconia material (IPS e.max ZirCAD Prime, Ivoclar Vivadent, Schaan, Liechtenstein), shade A2, and cemented to 2 composite core build-up materials using phosphate monomer containing resin-cement Multilink Automix (Ivoclar Vivadent, Schaan, Liechtenstein) were evaluated in this study. Based on a power analysis which showed that for α = 0.05, power = 0.8, 40 disc-shaped monolithic zirconia specimens and 40 disc-shaped composite core specimens were fabricated; 20 for each group (*n* = 20). Table 1 shows the materials used in this study, their composition, and manufacturers.

**Table1:** Materials used in the study as provided by the manufacturers

Brand	Material	Chemical composition			Manufacturer	batch number
IPS e.max ZirCAD Prime	Medium and high translucent zirconia (3Y-TZP and 5Y-TZP)	88.0 – 95.5% Zirconium oxide (ZrO2), > 4.5% – ≤ 7.0% Yttrium oxide (Y2O3), ≤ 5.0% Hafnium oxide (HfO2), ≤ 1.0% Aluminium oxide (Al2O3), ≤ 1.5% Other oxides			Ivoclar Vivadent AG (Schaan, Liechtenstein)	Z02B4J
Tetric N-Ceram	Nano-hybrid resin composite	Urethane dimethacrylate, ethoxylated Bis-EMA, Bis-GMA (18.8 wt%), barium glass filler, ytterbium trifluoride, mixed oxide (63.5 wt%), polymer (17 wt%), additives, catalysts, stabilizers, and pigments (0.7 wt%)			(Ivoclar Vivadent, Schaan, Liechtenstein)	U27917
MultiCore Flow	Self-cured core build-up composite with light-cured option	(Wt%) -Bis-GMA, urethane dimethacrylate, triethyleneglycol dimethacrylate highly dispersed silicon dioxide -Barium glass fillers, Ba-Al-fluorosilicate glass, highly dispersed silicone dioxide -Ytterbium trifluoride -Catalysts, stabilizers, pigments	Base 28.1 54.9 16.4 0.6	Catalyst 28.4 54.4 16.2 1.0	(Ivoclar Vivadent, Schaan, Liechtenstein)	Z001M5
Multilink Automix resin cement	two paste, self- curing adhesive resin cement	DMA and HEMA, adhesive monomer, barium glass filler, SiO2 filler, Ytterbium triflorite, accelarator and stabilisator and pigments			(Ivoclar Vivadent, Schaan, Liechtenstein)	K49940
Z-PRIME™ Plus	Primer	10-MDP, HEMA, Bis-GMA, ethanol			BISCO (Schaumburg, USA)	2100002784

*HEMA* 2-hydroxyethyl methacrylate, *10-MDP* 10-methacryoloyloxydecyl dihydrogen phosphate, *DMA* dimethacrylates, *Bis-GMA* bisphenol A-glycidyl methacrylate, *Bis-EMA* ethoxylated bisphenol A dimethacrylate

For the composite core material, 20 disc-shaped specimens of 10mm diameter and 4mm thickness for each group were prepared using a customized resin mold (Asiga DentaGUM, Sydney, Australia) that was designed with specific dimensions based on the digitized data (diameter = 10mm, thickness = 4mm) using CAD software (3shape Dental Software) and printed using 3D printer (Asiga 3D Printer, Sydney, Australia). For the first group, Tetric N-Ceram (Ivoclar Vivadent, Schaan, Liechtenstein), shade IVA, was applied in 2 increments for a total thickness of 4 mm into the resin mold until completely filled then carefully condensed with ST instrument with a plastic working end (OptraSculpt, Ivoclar Vivadent, Schaan, Liechtenstein) [38]. For the second group, MultiCore Flow (Ivoclar Vivadent, Schaan, Liechtenstein), shade Medium, was injected from its syringe into the resin mold in single bulk until completely filled using automatic mixing tips which allowed a homogeneous mixture [4]. A mylar strip and glass slide were placed on top of the mold to remove excess material, eliminate any voids and achieve a uniform surface finish. The materials were photo-polymerized using an LED curing light unit (Bluephase, Ivoclar Vivadent AG, Schaan, Liechtenstein) placed perpendicular on top of the glass plate surface for 40 s with an intensity of 1100 mW/cm2 and at a constant distance of 2mm*.* The specimens were removed from the mold, and excess composite was removed by fine polishing discs (Sof-Lex, 3M ESPE, St Paul, MN, USA) [39, 40].

The 40 disc-shaped zirconia specimens (2mm thickness,10mm diameter) were designed using CAD software (3Shape Dental Software) based on the digitized data and manufactured from partially sintered monolithic zirconia using a five-axis milling machine (Wieland Zenotec Select Hybrid, Ivoclar Vivadent AG, Schaan, Liechtenstein). The location of the cut was made through the middle layer of zirconia blocks. After separation from the blank, the samples were finished using finishing burs (Zolid Green-State Finishing Kit; Amann Girrbach AG). The enlarged green-state specimens were then sintered using a special furnace (Programat S1 1600, Ivoclar Vivadent AG, Schaan, Liechtenstein) according to the recommended manufacturer sintering protocol at a temperature of 1,500°C for about 4.5 h. The sintered specimens were then finished using finishing burs (Zolid Sinter-State Finishing Kit; Amann Girrbach AG) then abraded with 50 μm Al_2_O_3_-powder at 0.1 MPa pressure at constant distance of 10mm for 15 s (Dentify sandblaster, Engen, Germany), followed by ultrasonic cleaning in isopropanol bath for 5 min and air-drying for 15 s [27].

Two uniform coats of a phosphate-containing primer (Z-PRIME™ Plus; BISCO, Schaumburg, USA) were applied directly to the abraded zirconia specimens just before cementation, gently air-dried for 10 s, and light polymerized for 20 s [41].

Consequently, self-etching, dual-cure Multilink Automix resin-cement was used for cementation of the samples according to the manufacturers’ instructions. Resin-cement was applied from a syringe using automatic mixing tips to the intaglio surfaces of the zirconia specimens [42]. Thereafter, composite samples were bonded to the intaglio surfaces of zirconia specimens in a special device under a constant load of 750 g [43]. Excess cement was then removed using a disposable minibrush (Ivoclar Vivadent). Thereafter, the luting material was cured at a distance of 5mm for 20 s from each side. Oxygen protection gel (Air-Block Liquid Strip, Ivoclar Vivadent) was applied to the margins for 5 min to ensure total self-curing of the cement. Specimens were stored in distilled water at 37C° for 24 h.

All specimens were subjected to the biaxial flexural strength test at the universal testing machine (WDW-20; Jinan Testing Equipment IE Corporation, Jinan, China) according to ISO 6872:2008 for dental ceramics [44, 45]. The specimens were placed so that load was applied at the zirconia surface while the core surface of the specimen was at the bottom, mimicking the clinical situation.

For the piston-on-three-ball test, the sample holder was made of 3 hardened steel balls with a diameter of 3.2mm each. The steel balls were positioned at 120° apart forming an equilateral triangle on a support circle with a diameter of 10mm. The center of the specimens, which were placed upon the steel balls, and the center of the equilateral triangle were aligned coaxially. The load was applied centrally through a flat piston, with a diameter of 1.4mm, at a crosshead speed of 1mm/min until failure [44].

**Fig. 1:**
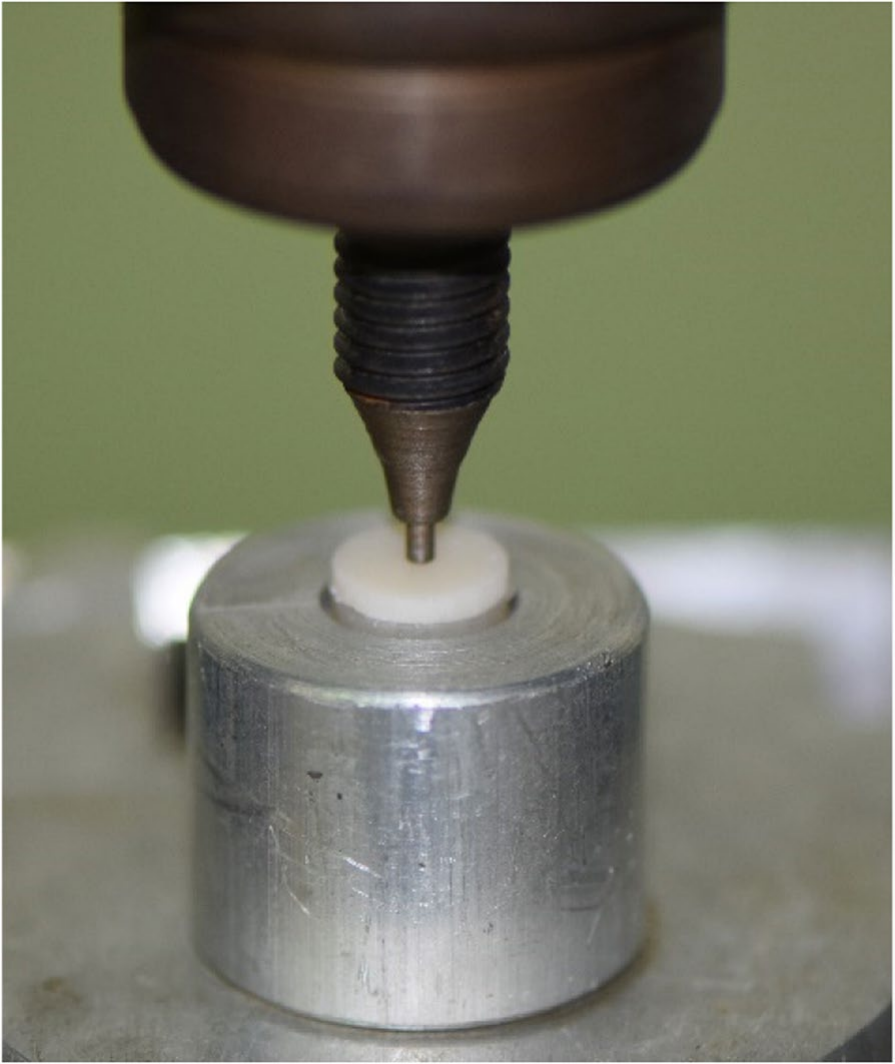
Load-to-failure test of samples using universal testing machine showing the piston touching the tested specimen

The load at fracture (P) was determined when a dramatic drop in the applied load occurred, associated with acoustic sound and values were recorded in Newton (N). Figure 1 shows one of the samples tested using the universal testing machine.

The biaxial flexural strength values in the zirconia and composite layers of the bilayered specimen were calculated using the following formulas derived by Hsueh et al. [46]:

$$\alpha 1=\frac{-\mathrm{E}1\left(\mathrm{z}-{\mathrm{z}}^{*}\right)\mathrm{P}}{8\uppi \left(1-\mathrm{v}1\right){\mathrm{D}}^{*}}\left\{1+2\mathrm{ln}\left(\frac{a}{c}\right)+\frac{1-v}{1+v}\left[1-\frac{{c}^{2}}{{2a}^{2}}\right]\frac{{a}^{2}}{{R}^{2}}\right\}$$

$$\alpha 2=\frac{-\mathrm{E}2\left(\mathrm{z}-{\mathrm{z}}^{*}\right)\mathrm{P}}{8\uppi \left(1-\mathrm{v}2\right){\mathrm{D}}^{*}}\left\{1+2\mathrm{ln}\left(\frac{a}{c}\right)+\frac{1-v}{1+v}\left[1-\frac{{c}^{2}}{{2a}^{2}}\right]\frac{{a}^{2}}{{R}^{2}}\right\}$$

where $\sigma 1$ is the maximum tensile stress in the composite core layer; $\sigma 2$ is the maximum tensile stress in the zirconia layer; P is the load at fracture; a is the radius of the supporting cycle; c is the radius of the piston; R is the radius of the disc; z is the interface between the layers in vertical cylindrical coordinate; t1 is the thickness of the composite core layer; t2 is the thickness of the zirconia layer; v1 is the Poisson ratio of composite core material; v2 is the Poisson ratio of zirconia material; z* is the neutral surface position; D* is the flexural rigidity; and v is the average Poisson ratio of the bilayer.

$${Z}^{*}=\frac{\frac{E1{t1}^{2}}{2\left(1-{v1}^{2}\right)}+\frac{E2{t2}^{2}}{2\left(1-{v2}^{2}\right)}+\frac{E2t1t2}{\left(1-{v2}^{2}\right)}}{\frac{E1t1}{\left(1-{v1}^{2}\right)}+\frac{E2t2}{\left(1-{v2}^{2}\right)}}$$

$${D}^{*}=\frac{E1{t1}^{3}}{3\left(1-{v1}^{2}\right)}+\frac{E2{t2}^{3}}{3\left(1-{v2}^{2}\right)}+\frac{E2t1t2\left(t1+t2\right)}{\left(1-{v2}^{2}\right)}-\frac{{\left[\frac{E1{t1}^{2}}{2\left(1-{v1}^{2}\right)}+\frac{E2{t2}^{2}}{2\left(1-{v2}^{2}\right)}+\frac{E2t1t2}{\left(1-{v2}^{2}\right)}\right]}^{2}}{\frac{E1t1}{\left(1-{v1}^{2}\right)}+\frac{E2t2}{\left(1-{v2}^{2}\right)}}$$

where E1 is the elastic modulus of the composite core layer and E2 is the elastic modulus of the zirconia layer. Elastic moduli and Poisson ratios of the tested materials were provided by the manufacturers. The elasticity moduli and the Poisson ratios of the IPS e.max ZirCAD Prime, Tetric N-Ceram, and MultiCore Flow were (210,0.3), (10.8,0.24), (7.5,0.28), respectively.

For this study, a = 5mm, c = 0.7mm, R = 5mm, t1 = 4mm, t2 = 2mm, z1 = 0, z2 = 2mm

$$v=\frac{v1t1+v2t2}{t1+t1}$$

In addition, Hsueh and Kelly provided analytical solutions for calculating the stresses at the outer surfaces of the discs, $\sigma T$ (top) and $\sigma B$(bottom) [47]:

$$\sigma T=\frac{\left(\frac{6E2M}{\left(1-V2\right)}\right)\left[\frac{E1{t1}^{2}}{\left(1-v1\right)}+\frac{E2{t2}^{2}}{\left(1-v2\right)}+\frac{2E1t1t2}{\left(1-v1\right)}\right]}{{\left[\frac{E1{t1}^{2}}{\left(1-v1\right)}+\frac{E2{t2}^{2}}{\left(1-v2\right)}\right]}^{2}+\frac{4E1E2t1t2\left({t1}^{2}+t1t2+{t2}^{2}\right)}{\left(\left(1-v1\right)\left(1-v2\right)\right)}}$$

$$\sigma B=\frac{\left(\frac{-6E1M}{\left(1-V1\right)}\right)\left[\frac{E1{t1}^{2}}{\left(1-v1\right)}+\frac{E2{t2}^{2}}{\left(1-v2\right)}+\frac{2E1t1t2}{\left(1-v1\right)}\right]}{{\left[\frac{E1{t1}^{2}}{\left(1-v1\right)}+\frac{E2{t2}^{2}}{\left(1-v2\right)}\right]}^{2}+\frac{4E1E2t1t2\left({t1}^{2}+t1t2+{t2}^{2}\right)}{\left(\left(1-v1\right)\left(1-v2\right)\right)}}$$

where M is the biaxial bending moment.

$$M=\frac{-\mathrm{P}}{8\uppi }\left\{\left(1+v\right)\left[1+2\mathrm{ln}\left(\frac{a}{c}\right)\right]+\left(1-v\right)\left[1-\frac{{c}^{2}}{{2a}^{2}}\right]\frac{{a}^{2}}{{R}^{2}}\right\}$$

Furthermore, stresses at composite and zirconia interfaces, $\sigma 1 stress$ and $\sigma 2 stress$, respectively, were calculated according to the following equations:

$$\sigma 1=\frac{E1\left(1-v2\right)t1\sigma T}{E2\left(1-v1\right)\left(t1+t1\right)}+\frac{t2\sigma B}{t1+t2}$$

$$\sigma 2=\frac{t1\sigma T}{t1+t2}+\frac{E2\left(1-v1\right)t2\sigma B}{E1\left(1-v2\right)\left(t1+t2\right)}$$

Analytical data calculations were carried out using the SPSS version 26.0 (SPSS, Chicago, IL, USA) statistical program. The results were statistically analyzed using the independent t-test (parametric inferential statistics). All data were subjected to Levene’s test of homogeneity of variance (α < 0.05) following the assumption of equal variance. A statistical significance was determined given a p-value less than 0.05 (2-tailed). The mean biaxial flexural strength values of the 2 zirconia groups, the mean biaxial flexural strength values of the 2 composite core materials, the mean stresses at the top and bottom of the bilayered specimens for each group, and the mean interfacial stresses at the zirconia and composite core layers for each group were calculated, analyzed, and compared for any statistically significant differences. In addition, the biaxial flexural strength values for each zirconia group were also analyzed using Weibull statistics (Weibull +  + version 6, Reliasoft Corp., Tuczon, AZ, USA) to evaluate the flexural strength’s reliability and to estimate characteristic strength (σo) as well as the Weibull modulus (m) according to the following equation:

*Pf* = 1 − *exp* $${\left[\frac{\sigma }{\sigma 0 }\right]}^{m}$$

Where *Pf* is the probability of failure, σ is the fracture strength, σ0 is the characteristic strength, and m is the Weibull modulus.

## Results

**Fig. 2:**
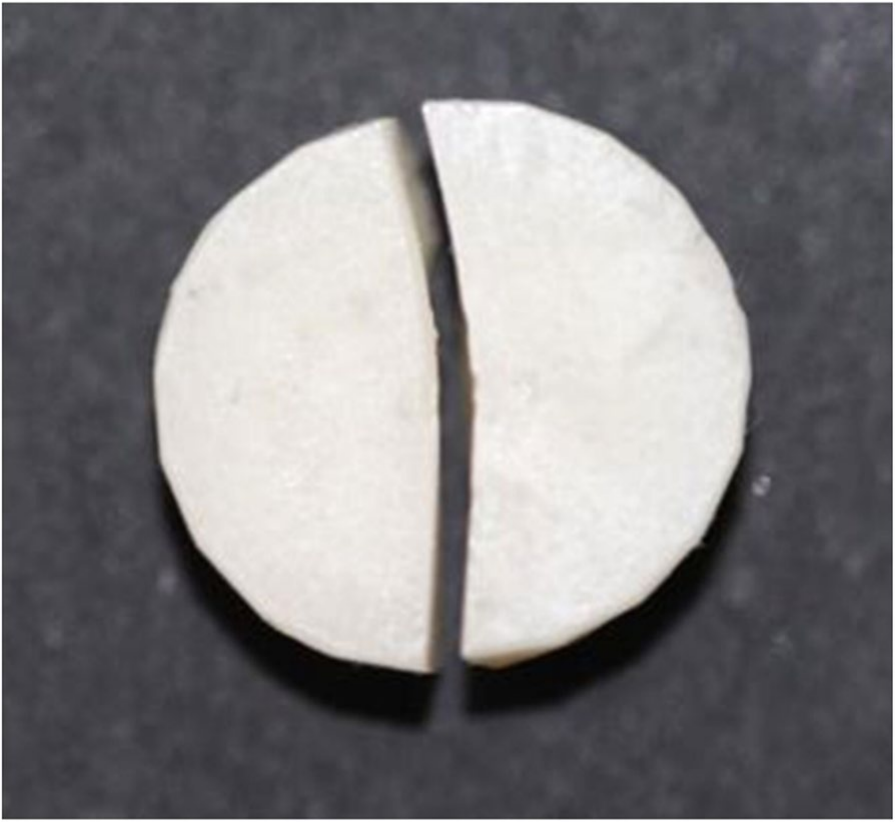
The fractured bilayered composite-zirconia specimen

The visual analysis of the fractured specimens revealed 1 type of failure for all bilayered specimens in both groups: complete fracture, through the zirconia and composite core layers. An example of the fractured specimen is shown in Fig. 2. All the tested composite-zirconia specimens were fractured into 2 or 3 segments with the 2 cemented layers remaining in contact without any sign of delamination or separation. Table 2 demonstrates the calculated (P) ± standard deviation (SD) in Newton (N) for both tested groups.

**Table2:** The mean load at fracture, in Newtons, and standard deviations (SD) for both experimental groups

**Material**	Mean P(SD)
**Zirconia-Tetric N-Ceram**	1582 (220.20)
**Zirconia-MultiCore Flow**	1348 (268.24)

The Kolmogorov–Smirnov test revealed no deviation from the normal distribution and data were analyzed parametrically (*p* > 0.05). No statistically significant difference in the biaxial flexural strength values of the 2 zirconia groups cemented was found (*p* = 0.27). Higher Weibull modulus value was obtained for the Zirconia-Tetric N-Ceram compared to the Zirconia-MultiCore Flow. Three-point flexural strength results and characteristic strength values for the 2 tested zirconia groups were in the same order. The Weibull statistical analysis of the biaxial flexural strength data is summarized in Table 3.

**Table 3:** Weibull statistical results

Material	Weibull modulus (m)	Characteristic strength (MPa) (σ0)
**Zirconia-Tetric N-Ceram**	8.54	844.6
**Zirconia-MultiCore Flow**	5.89	803.8

Comparing the biaxial flexural strength values of the 2 tested composite core materials, Tetric N-Ceram had a significantly higher biaxial flexural strength values compared to MultiCore Flow (*p* < 0.001). Furthermore, for both tested groups, the stress values at the top layers were higher than the stress values at the bottom layers and the interfacial stresses at the zirconia layers were higher than the interfacial stresses at the composite core layers.

Although no statistically significant differences were found in the interfacial stresses at the composite core layers between the 2 tested groups (*p* = 0.12), the Zirconia-MultiCore Flow group had significantly higher stresses values at the top layer and at the interfacial zirconia layer ((*p* < 0.001), *p* = 0.008 respectively) but significantly lower stress values at the bottom layer (*p* < 0.001).

**Fig. 3:**
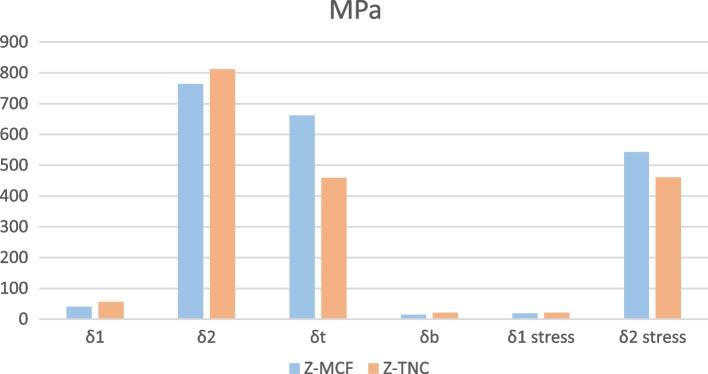
Histogram showing the differences in biaxial flexural strength of the composite layers (${{\sigma}}1$), biaxial flexural strength of the zirconia layers (${{\sigma}}2$), stresses at the top (${{\sigma}}{{t}}$), bottom (${{\sigma}}{{b}}$), and the interfacial stress values at the composite (${{\sigma}}1{ }{{s}}{{t}}{{r}}{{e}}{{s}}{{s}}$) and at the zirconia (${{\sigma}}2{ }{{s}}{{t}}{{r}}{{e}}{{s}}{{s}}$) layers between the two tested groups

These results are summarized in Table 4 and Fig. 3.

**Table 4:** Mean and standard deviation (SD) of the biaxial flexural strength of the two composite core materials ($\sigma 1$) and the two zirconia groups ($\sigma 2$), the stress values at the top ($\sigma t$) and at the bottom ($\sigma b$) for both groups, the interfacial stress values at the composite ($\sigma 1 stress$) and at the zirconia ($\sigma 2 stress$) layers for both groups, and the independent t-test statistical results

	$\mathbf{m}\mathbf{e}\mathbf{a}\mathbf{n}(\mathbf{S}\mathbf{D})$ **for Zirconia-Tetric N-Ceram (Mpa)**	$\mathbf{m}\mathbf{e}\mathbf{a}\mathbf{n}(\mathbf{S}\mathbf{D})$ **for Zirconia-MultiCoreFlow (Mpa)**	**t-value**	***p*-value**	**Mean difference**	**95% confidence interval of the difference**	
						**lower**	**Upper**
${\varvec{\sigma}}\boldsymbol{1}$	55.86 (7.77)	40.78 $\pm$ 8.14	5.917	< 0.001	15.082	9.918	20.246
${\varvec{\sigma}}\boldsymbol{ 2}$	811.60 (112.94)	763.64 (152.47)	1.120	= 0.270	47.963	38.785	134.710
${\varvec{\sigma}}\boldsymbol{ }{\varvec{t}}$	-458.65 (63.82)	-662.24 (132.23)	6.173	< 0.001	203.597	136.774	270.420
${\varvec{\sigma}}\boldsymbol{ }{\varvec{b}}$	20.86 2.90	14.84 (2.96)	6.407	< 0.001	6.019	4.116	7.923
${\varvec{\sigma}}$ **1 stress**	20.79 (2.89)	19.07 (3.81)	1.592	= 0.120	1.719	0.468	3.906
${\varvec{\sigma}}2$ $\mathbf{s}\mathbf{t}\mathbf{r}\mathbf{e}\mathbf{s}\mathbf{s}$	460.21 (64.04)	542.03 (108.23)	2.854	= 0.008	81.82	23.189	140.442

## Discussion

The present study investigated the biaxial flexural strength of 2 different composite core materials (Tetric N-Ceram and MultiCore Flow) and their influence on the biaxial flexural strength of monolithic multilayered strength-gradient zirconia restorations. The results showed no significant effect of the composite core materials on the biaxial flexural strength of the zirconia specimens (*p* = 0.270), hence the first null hypothesis was accepted. However, Tetric N-Ceram had significantly higher biaxial flexural strength than MultiCore Flow (*p* < 0.001) and therefore the second null hypothesis was rejected.

The results of this study showed that the tested monolithic multilayered zirconia material had sufficiently high biaxial flexural strength values allowing its use for the construction of three-unit fixed partial dentures including molar restorations, as the minimum ISO value is 500 MPa [24].

The mean biaxial flexural strength for the zirconia cemented to Tetric N-Ceram was 811.60 Mpa while for the zirconia cemented to MultiCore Flow was 763.64 Mpa in our study, which were statistically similar, indicating that variation in the modulus of elasticity and curing method of the underlying core materials might have no effect on the biaxial flexural strength of the overlying zirconia restorations. Similar findings were previously reported by Azer et al. in 2001, while Abdelaziz et al. 2018 reported that composite resins with higher strengths and moduli of elasticity resulted in superior fracture resistance for the overlaying glass ceramic crowns [3, 17]. This disagreement can be attributed to the smaller difference in elastic moduli between the 2 composites used in our study compared to Abdelaziz et al., or due to the difference in all-ceramic material examined [3].

The Weibull analysis was carried out on the biaxial flexural strength values since it provides consistent data about the fracture of brittle dental materials [29]. Zirconia-Tetric N-Ceram revealed a higher Weibull characteristic strength value than Zirconia-MultiCore Flow and the values were comparable to previous studies, indicating superior structural reliability of the material [27, 31]. The high fracture load recorded for the 2 tested zirconia groups might be associated with the high-strength 3Y-TZP core and its increased content of tetragonal crystals which offer a strengthening effect because of the transformation toughening [27]. Comparable values were found by Rosentritt et al. 2022 and Schonhoff et al. 2021 who also reported that fracture load for the specimens taken from the middle layer of IPS e.max ZirCAD Prime was higher than those taken from the uppermost incisal layer which is composed of 5Y-TZP [30, 31]. Therefore, in our study, samples were cut from the middle layer of zirconia blanks.

When the prepared bilayered specimen is composed of 2 distinct layers with different moduli of elasticity, large stresses usually emerge. Two different stress zones arise through the thicknesses of the discs: the compressive force zone at the top layer and the tensile force zone at the bottom layer which is more critical since it induces more problems than compressive stresses for brittle materials as ceramics [5, 36]. Furthermore, due to the modulus mismatch between the 2 cemented layers, interfacial stresses also arise [36, 44]. The present study assessed the stresses through the whole thickness of the bilayered specimen which included the mean values of stress at the top, interfacial, and bottom layers. For both groups, the stresses that occurred at the zirconia layers, at the top surfaces, and at the interfaces of the zirconia layer were higher than those that occurred at the composite layers, at the bottom surfaces, and at the interfaces of the composite layer. Furthermore, the results showed that Zirconia-MultiCore Flow had significantly higher stresses at the top layer but significantly lower stresses at the bottom layer ($\sigma t=662.24 , \sigma b= 14.84$) compared to the Zirconia-Tetric N-Ceram ($\sigma t=458.65 , \sigma b=20.86$) (*p* < 0.001), *p* < 0.001 respectively). This can be attributed to the different chemical composition of Tetric N-Ceram and MultiCore Flow along with the lower modulus of elasticity, and stiffness of MultiCore Flow compared to the Tetric N-Ceram. Although no statistically significant differences were found in the interfacial stresses at the composite layers between the 2 tested groups (*p* = 0.12), variation in the chemical composition and the mechanical properties of Tetric N-Ceram and MultiCore Flow resulted in higher interfacial stress at the zirconia layer in the Zirconia-MultiCore Flow (*p* = 0.008) [36, 44].

All specimens in our study exhibited 1 type of failure which was complete fracture through the entire bilayered specimen that seemed to act as a homogenous material without any sign of delamination or separation. This immediate fracture occurs when interfacial toughness is greater than the flexural stresses in the tension surface at failure and because the strengths at the bottom surface of the bilayered specimens ($\sigma$ b) showed lower values compared to those at the interface of the zirconia layer ($\sigma stress 2$) [44].

Core buildup materials used to restore fractured, broken, and endodontically treated teeth should be of satisfactory strength to resist stresses during function and mastication [17]. The core build‑up materials investigated in our study were resin composites with variable polymerization processes, resin formulas, and filler characteristics and are commonly used for direct restorations in practice [3]. The results of our study showed that biaxial flexural strength of Tetric N-Ceram (55.86 $\pm$ 7.77 Mpa) was significantly higher than that of MultiCore Flow (40.78 $\pm$ 8.14 Mpa). This difference might be attributed to variations in the chemical composition, type of resin, type of inorganic filler, and size and content of filler particles between the 2 composite materials [9]. Increasing the volume of the filler and the level of filler weight of the composite materials was reported to result in an increase in the strength of the materials [4, 6, 11]. This might explain the results of our study, as Tetric N-Ceram has a higher filler volume (63.5% vol) compared to MultiCore Flow (54.4% vol). Rüttermann et al. reported that, in addition to filler loading, filler type and content might have significant correlation to the flexural strength values of the material [5]. Furthermore, higher filler content enhances the modulus of elasticity resulting ultimately in greater resistance to fracture and higher flexural strength [40]. This can be confirmed in our study, as the Tetric N-Ceram (*E* = 10.8) has a higher modulus of elasticity compared to the MultiCore Flow (E = 7). However, this disagrees with the results of some studies that found that variations in the moduli of elasticity had no influence on fracture resistance and flexural strength as multiple factors might also contribute to the overall strength of the material including the resin matrices, different types of fillers, or filler size and their distribution [13, 39]. Warangkulkasemkit and Pumpaluk 2019 suggested that the lower value of biaxial flexural strength of MultiCore Flow might be related to the fact that it contains barium glass and silicon dioxide fillers with no zirconia particles in its composition [4]. On the other hand, the presence of nano-sized filler particles along with high molecular weight monomers in the composition of the Tetric N-Ceram might contribute to its higher biaxial flexural strength value [9, 15].

One of limitations of our study was the absence of tooth substrate. However, previous studies proved the reliability of using the piston-on-three-ball test for studying the biaxial flexural strength of brittle dental materials such as ceramics [34]. Furthermore, future studies should focus on investigating the effect of aging and thermocycling in an environment that mimics the oral cavity along with the need for long-term clinical studies to validate the results obtained in this study. In addition to that, this investigation used only one type of multilayered monolithic zirconia. Forthcoming research including various brands and types of zirconia aiming to compare this novel multilayered zirconia with the conventional zirconia types is required.

## Conclusions

Within the limitations of this in vitro study, the following conclusions can be withdrawn:

1. Tetric N-Ceram composite core material have superior biaxial flexural strength than MultiCore Flow material.
2. The type of core material used to restore endodontically treated teeth does not affect the biaxial flexural strength values of the overlying zirconia restorations.
3. IPS e.max ZirCAD Prime has high biaxial flexural strength value allowing its use for the construction of anterior and posterior indirect restorations.

## Data Availability

This article has all the data that were collected or analyzed during this study.

## Abbreviations

3 Y-TZP: 3 Mol % Yttria-Stabilized Tetragonal Zirconia Polycrystals
4 Y-TZP: 4 Mol % Yttria-Stabilized Tetragonal Zirconia Polycrystals
5 Y-TZP: 5 Mol % Yttria-Stabilized Tetragonal Zirconia Polycrystals
CAD/CAM: Computer-Aided Design/Computer-Aided Manufacturing
ISO: The International Organization for Standardization
N: Newton
SD: Standard Deviation
